# A Pathogenic Role for FcγRI in the Immune Response against Chlamydial Respiratory Infection

**DOI:** 10.3390/microorganisms11010039

**Published:** 2022-12-22

**Authors:** Jiajia Zeng, Shuaini Yang, Ruoyuan Sun, Yuqing Tuo, Lu Tan, Hong Zhang, Yongci Zhang, Xuchun Che, Tingsha Lu, Xuejun Zhang, Hong Bai

**Affiliations:** Key Laboratory of Immune Microenvironment and Disease (Ministry of Education), Department of Immunology, School of Basic Medical Sciences, Tianjin Medical University, Tianjin 300070, China

**Keywords:** chlamydial infection, FcγRI, Th1 response, macrophage, polarization, inflammation

## Abstract

FcγRI is an important cell surface receptor reported to be involved in multiple immune responses, although it has not yet been extensively studied in intracellular bacterial infections. Here, using a mouse model of *C. muridarum* respiratory infection, we were able to determine how FcγRI regulates the host resistance against chlamydial invasion. According to our findings, the chlamydial loads and pulmonary pathology were both reduced in FcγRI deficient (Fcgr1^−/−^) animals. Being infected, monocytes, macrophages, neutrophils, DCs, CD4^+^/CD8^+^ T cells, and effector Th1 subsets displayed increased FcγRI expression patterns. Altered infiltration of these cells in the lungs of Fcgr1^−/−^ mice further demonstrated the regulation of FcγRI in the immune system and identified Th1 cells and macrophages as its target cell populations. As expected, we observed that the Th1 response was augmented in Fcgr1^−/−^ mice, while the pro-inflammatory M1 macrophage polarization was constrained. These findings might indicate FcγRI as a potential regulator for host immunity and inflammatory response during chlamydial infection.

## 1. Introduction

*Chlamydia trachomatis* (*C. trachomatis*) is a Gram-negative obligate intracellular bacterium that is commonly responsible for blinding trachoma, sexually transmitted diseases, pelvic inflammatory disease, and neonatal pneumonia [1,2]. Although chlamydial infections are treatable with antibiotics, the asymptomatic and relapse characteristics complicate infection eradication and predispose the host to chronic inflammatory pathology [3]. The host defense against *C. trachomatis* infection involves both the innate (including infected epithelial cells, NK cells, neutrophils, macrophages, dendritic cells, and inflammatory factors) and adaptive immune responses (including B cells, CD4^+^ and CD8^+^ T cell responses) [4,5]. Several studies have demonstrated CD4^+^ T cells as the essential lymphocyte population. After infection, naïve CD4 T cells differentiate into type 1 T helper (Th1), Th2, Th17, T follicular helper (Tfh), or regulatory T cells (Treg) lineages, CD4^+^ Th1 cells are the major effector lineage that mediates chlamydia clearance via interferon-gamma (IFN−γ) secretion [3,6].

In mouse research, equipped on myeloid, B, and natural killer (NK) cells, the receptors for the Fc portion of immunoglobulins (FcRs) endow these target cells with the ability to interact directly with IgM, IgG, IgA, and/or IgE classes of immunoglobulins [7]. Fcγ-receptors (FcγRs) are receptors specific to the IgG Fc portion and can be functionally divided into activating (FcγRI, FcγRIII, and FcγRIV) and inhibitory (FcγRIIB) receptors, which transmit signals via immunoreceptor tyrosine-based activation (ITAM) or inhibitory motifs (ITIM) [8]. Among them, FcγRI is the only activating receptor that binds IgG2a with high affinity and has shown to be constitutively expressed on the surface of monocytes, macrophages, and monocyte-derived dendritic cells (moDCs), as well as inducible on neutrophils under inflammatory conditions [9,10,11]. In recent years, many in vivo observations in FcγRI-deficient mice suggest a pleiotropic regulatory role of FcγRI in the immune system, as described in several infections and inflammatory diseases such as Arthus reaction, arthritis, *Bordetella pertussis* infection, and Porcine reproductive and respiratory syndrome virus (PRRSV) infection [12,13,14]. Despite this, no studies have linked FcγRI with respiratory tract immunity against chlamydial infection.

In the present investigation, using the FcγRI gene-knockout (Fcgr1^−/−^) mice and the *C. trachomatis* mouse pneumonitis biovar (*C. muridarum*), we examined the impact of FcγRI on innate and adaptive immunity in a well-established mouse model of pulmonary *C. muridarum* infection. In contrast to WT controls, our findings revealed that Fcgr1^−/−^ mice exhibit relieved chlamydial loads and pulmonary pathology, which could be attributed to the elevated Th1 response and reduced M1 macrophage polarization. This study is the first to characterize the role of FcγRI during chlamydial respiratory infection and identify FcγRI as a potential therapeutic target for *C. trachomatis* infectious illness.

## 2. Materials and Methods

### 2.1. Mice

In this study, we used 6–8 weeks-old female mice (weighing 18–20 g). C57BL/6 mice (wild-type, WT) were purchased from Huafukang Biotechnology Co., Ltd. (Beijing, China). Fcgr1^−/−^ mice (C57 BL/6 background) were purchased from Shanghai Model Organisms Center (Shanghai, China). Mice were maintained in specific pathogen-free conditions at Tianjin Medical University (Tianjin, China). The animals were randomly divided into different groups (n = 3–6 per group), and all the mice experiments were repeated at least 3 times. All procedures involving animals were reviewed and approved by the Animal Ethical and Welfare Committee (AEWC) of Tianjin Medical University (number of animal permit: SYXK: 2016-0012, approval date: 7 March 2018).

### 2.2. Mouse Model of C. trachomatis Respiratory Infection

The *C. trachomatis* mouse pneumonitis biovar, also known as *Chlamydia muridarum* (*C. muridarum*), was presented by Dr. Xi Yang (The University of Manitoba, Canada). *C. muridarum* organisms were cultivated, purified, and quantified as described [15]. Mice were anesthetized with isoflurane and 40 μL sucrose phosphate glutamic acid (SPG) buffer containing 1 × 10^3^ inclusion forming units (IFUs). *C. muridarum* were intranasally inoculated to induce chlamydial infection, with the uninfected mice (0 d) inoculated with 40 μL SPG buffer as a control.

### 2.3. IFUs Detection

The lung tissues were homogenized aseptically in the SPG buffer, acquired lung homogenates were centrifuged at 4 °C for 30 min, and diluted supernatants were added into a confluent monolayer of HeLa cells and incubated at 37 °C for 2 h. After being cultured for 40–48 h, media were removed and infected cells were fixed with methanol for 10 min. The cells were then stained with the Chlamydia LPS antibody (Invitrogen, Waltham, MA, USA), and HRP-conjugated goat anti-mouse IgG secondary Abs (Solarbio, Beijing, China) and developed with the substrate (4-chloro-1-naphthol; Solarbio). The inclusion bodies were counted under the microscope [×200] to calculate IFUs per sample.

### 2.4. Histology Analysis and Semi-Quantitative Pathological Scoring

For histopathological analyses, *C. muridarum*-infected lung tissues were fixed in 4% paraformaldehyde (PFA) (Life-iLab, Shanghai, China) and routinely embedded in paraffin, sectioned (5 μm), and stained with hematoxylin and eosin (H&E) according to the manufacturer’s instructions (Solarbio). The pathological changes were evaluated by the semi-quantitative histology score in a blinded manner as described previously [16]. Briefly, the inflammatory grades were scored as: score 0, normal; score 1, moderate and limited inflammatory granuloma with less than 25% cell infiltration and no obvious infiltration in adjacent alveolar septa or air space; score 2, moderate interstitial pneumonia with 25–50% cell infiltration and septal congestion and septal edema; score 3, inflammatory cells infiltrated into perivascular, peribronchiolar, alveolar septa and air space, accounting for 50–75% of the area; score 4, over 75% of the lung area infiltrated with inflammatory cells.

### 2.5. Immunofluorescent Staining

The sterile-separated lung tissue was fixed in 4% PFA, dehydrated with 15% and 30% sucrose solutions and Tissue-Tek O.C.T. Compound (SAKURA) embedded, and 8 μm lung cryosections were cut. The lung sections were blocked with 10% goat serum at room temperature for 1 h, then incubated with anti-F4/80 antibody [BM8] (1:100, Abcam, Cambridge, UK) at 4 °C overnight. The primary antibody was removed and Goat Anti-Rat IgG H&L (Alexa Fluor 488)-conjugated secondary antibody (1:500, Abcam) was added at room temperature for 1 h in the dark. Finally, DAPI Fluoromount (SouthernBiotech, Alabama, USA) was added and images were acquired under a fluorescent microscope [×200].

### 2.6. Lung and Spleen Single Cells Preparation

These single-cell suspensions were prepared as described previously [10,17]. Briefly, sterile-isolated lung tissue from mice at different time points post infection was minced and incubated with collagenase XI (Sigma-Aldrich, Ronkonkoma, N.Y., USA) at 37 °C for 55 min. Tissue fibers and erythrocytes were successively removed by 35% Percoll (GE Healthcare, Chicago, Illinois, USA) and ACK Lysis buffer (Tris-NH_4_Cl). Sterile-isolated spleen tissue was directly ground and filtered through 70-μm cell strainers, and erythrocytes were lysed using the ACK lysing buffer. Single cells were washed and resuspended in a complete RPMI-1640 medium (RPMI-1640 supplemented with 10% heat-inactivated FBS, 100 U/mL penicillin, and 0.1 mg/mL Streptomycin) for counting and flow cytometry analysis.

### 2.7. Antibodies and Flow Cytometry

The monocytes and neutrophils were subsequently stained with the following surface antibodies: anti-CD45-PerCP-Cy5.5, anti-CD11b-FITC, anti-MHCII-PE, anti-Ly6G-APC, and anti-Ly6C-PE-Cy7. Macrophages were stained with surface antibodies, including: anti-CD45-PerCP-Cy5.5, anti-F4/80-APC, anti-CD80-PE-Cy7, anti-CD86-PE, anti-MHCII-PE, and anti-CD206-PE-Cy7; intracellular antibodies IL−10-PerCP-Cy5.5 and TGF−β-PE; and intranuclear antibodies iNOS-PE. DCs were stained with antibodies including anti-CD45-APC-Cy7, anti-CD11c-APC, and anti-MHCII-PerCP-Cy5.5. T cells were stained with surface antibodies, including: anti-CD3-FITC, anti-CD4-APC, and anti-CD8-PerCP; intracellular antibodies including anti-IFN−γ-PE-Cy7, anti-IL−17A-PE, and anti-IL−4-PE; and intranuclear antibodies anti-FoxP3-PE and anti-Ki−67-PE. Anti-FcγRI-APC was used as a surface antibody. All antibodies are purchased from BioLegend (San Diego, CA, USA) or BD Biosciences (BD Biosciences, Sparks, MD, USA).

Single-cell suspensions were resuspended in FACS buffer (PBS with 2% FBS) and incubated with Fc receptor block Abs (anti-CD16/CD32 monoclonal Abs; eBioscience, San Diego, CA, USA) at 4 °C for 20 min in the dark. For cell-surface staining, cells were stained with conjugated antibodies specific for cell surface markers at 4 °C for 30 min in the dark. For intracellular cytokine staining, cells were stimulated with PMA (50 ng/mL), ionomycin (1 μg/mL, Sigma–Aldrich), and brefeldin A (10 μg/mL, BioLegend) at 37 °C for 5–6 h. Following a blocking step similar to cell-surface staining, cells were stained first for surface antigens and then fixed with Fixation buffer (BioLegend). Fixed cells were washed and permeabilized with 1 × Intracellular Staining Perm Wash Buffer (BioLegend) followed by incubation with anti-IFN−γ, anti-IL−4, or anti-IL−17A mAb for 30 min at room temperature. For intranuclear iNOS staining, cells were stained for surface antigens, fixed, permeabilized with 1 × Intracellular Staining Perm Wash Buffer, and stained for anti-iNOS for 30 min at room temperature. For intranuclear Foxp3 and Ki-67 staining, cells were stained for surface antigens, fixed and permeabilized with 1 × Fix/Perm Buffer (BD Biosciences) at 4 °C for 50 min, washed by 1 × Perm/Wash Buffer, and incubated with anti-Foxp3 or anti-Ki-67 at room temperature for another 50 min. Finally, cells were suspended with FACS buffer, detected using a FACSCanto II flow cytometer (BD Biosciences), and analyzed using the FlowJo V10 software.

### 2.8. RNA Extraction and Quantitative Real-Time PCR (qPCR)

The lung tissues were isolated and homogenated aseptically, the total RNA was extracted by TRIzol reagent (Invitrogen), and the reverse transcription was performed using the cDNA Synthesis SuperMix (TransGen Biotech, Beijing, China) according to the manufacturer’s instructions. The qPCR analysis was performed using the RealStar Fast SYBR qPCR Mix (GenStar, Beijing, China) and proceeded on Light Cycler 96 (Roche, Basel, Switzerland). The mRNA expression level of target genes was presented as the “fold change” relative to that of control samples, with fold changes calculated by the 2^−ΔΔCt^ method using the mouse β-actin gene as an endogenous control. Primer sequences were shown in Supplementary Table S1.

### 2.9. Statistical Analysis

Data are shown as mean ± SD and were analyzed with GraphPad Prism version 9. Differences between two different groups were assessed using two-way ANOVA followed by Šidák’s multiple comparisons test, and differences between multiple groups were analyzed by one-way ANOVA followed by Dunnett’s multiple comparisons test. All *p* values < 0.05 were considered significant.

## 3. Results

### 3.1. FcγRI Participates in and Regulates the Immune Response against Chlamydial Respiratory Infection

**Figure 1:**
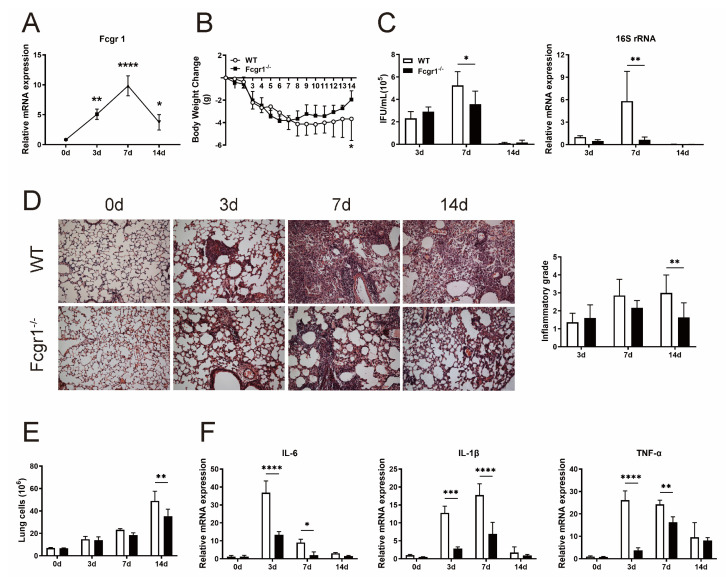
Increased resistance of FcγRI deficient (Fcgr1^−/−^) mice to *Chlamydia muridarum* (*C. muridarum*) respiratory infection. (**A**) C57 BL/6 mice were intranasally infected with 1 × 10^3^ inclusion forming units (IFUs) *C. muridarum* and sacrificed on different days post infection (p.i.). Total RNA extracted from the lung tissue was assayed for Fcgr1 mRNA expression by quantitative real-time PCR (qPCR). (**B**–**F**) Following intranasal infection, the wild-type (WT) and Fcgr1^−/−^ mice were daily monitored for body weight changes (**B**). (**C**) The chlamydial loads in the lung at days 3, 7, and 14 p.i. were determined by the levels of IFUs (**left**) and 16S rRNA mRNA expression (**right**). (**D**) The infected lung sections were stained with H&E and observed in × 200 magnification under light microscopy, the inflammatory grades were evaluated as described in the *Materials and methods*. (**E**) The entire number of lung cells of WT and Fcgr1^−/−^ mice. (**F**) Total RNA extracted from the lung tissue was assayed for IL−6, IL−1β, and TNF−α mRNA expression by qPCR. Data are represented as means ± SD from n = 3–5 per group, representative of one of three independent experiments. Statistical significances of differences are determined by one-way ANOVA (**A**) and two-way ANOVA (**B**–**F**). * *p* < 0.05, ** *p* < 0.01, *** *p* < 0.001, **** *p* < 0.0001.

To determine the participation of FcγRI in chlamydial infection, we employed our established model of chlamydial respiratory infection by intranasal injection of *C. muridarum* into C57 BL/6 mice as previously described [10,16]. As the 3 days post-infection (3 d p.i.), 7 d p.i. and 14 d p.i. represent different infectious periods, the lungs were harvested, and increased expression levels of Fcgr1 mRNA were detected as the infection progressed (Figure 1A), indicating the involvement of FcγRI in response to chlamydial infection.

We next compared the immunopathological consequences between WT and Fcgr1^−/−^ mice to evaluate the impact of FcγRI. As shown in Figure 1B–D, chlamydial invasion induced body weight losses with pulmonary chlamydial loads, as the IFUs and 16S rRNA expression correspond to intracellular live bacteria. As the infection progressed to the late period (14 d p.i.), host immunity effectively cleared the replicated chlamydia; however, it left visible pulmonary pathology characterized by massive inflammatory cell infiltration, destruction of pulmonary alveoli, and lung consolidation. Deficiency of FcγRI resulted in less body weight loss, lower IFUs, and reduced 16S rRNA expression, demonstrating greater inhibition of bacterial replication compared with WT controls. To further identify the link between FcγRI deficiency and chlamydia-induced pathogenesis, histologic analysis was conducted and alleviative pulmonary pathology with lower inflammatory grades was detected in the Fcgr1^−/−^ mice than in WT controls (Figure 1D). The fewer lung cell numbers and lower mRNA expression of pro-inflammatory cytokines IL−6, IL−1β, and TNF−α also indicated the constrained inflammatory response in Fcgr1^−/−^ mice (Figure 1E,F). Collectively, these results supported the pathogenic role of FcγRI by suppressing chlamydial clearance and exacerbating inflammatory pathological response against chlamydial infection.

### 3.2. Chlamydial Respiratory Infection Induces Increased Expression Pattern of FcγRI in the Innate and Adaptive Immune System

**Figure 2:**
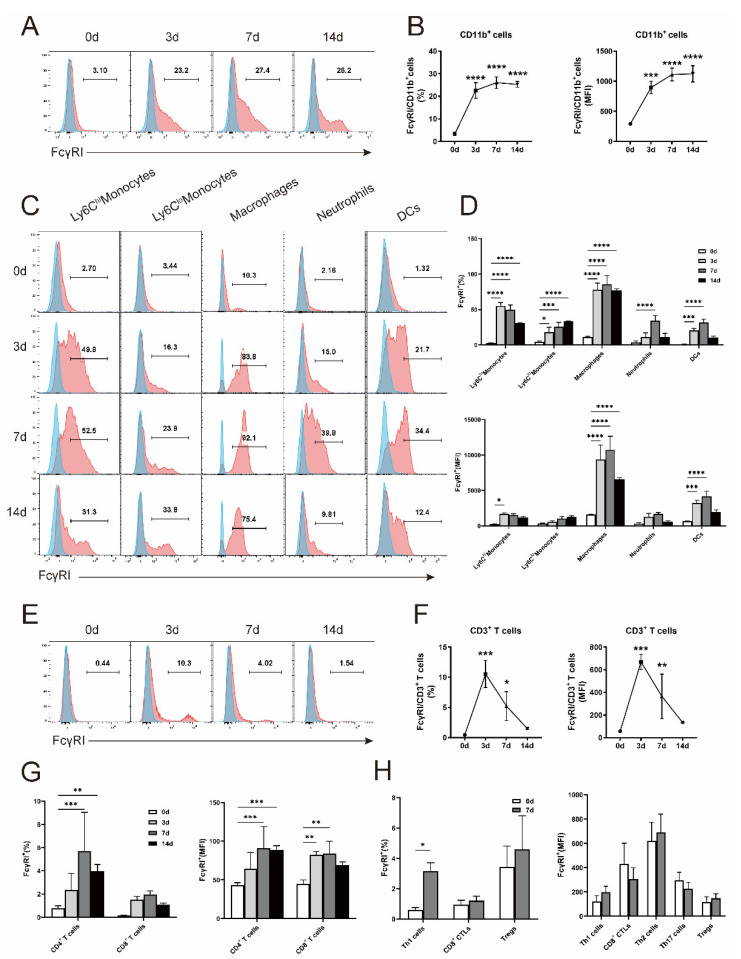
Increased expression of FcγRI in innate and adaptive immune cells after infection. The WT mice were intranasally infected and euthanized at days 0, 3, 7, and 14 p.i., lung single cells were prepared and flow cytometry was performed to determine the FcγRI expressions (red) with fluorescence minus one (FMO) control (blue) on gated cells as described in Supplementary Figure S1. Representative flow cytometric images (**A**, **C**, and **E**), the percentages and mean fluorescence intensity (MFI) of FcγRI^+^ cells on the CD11b^+^ cells (**B**), Ly6Chi monocytes, Ly6C^lo^ monocytes, macrophages, neutrophils, and DCs (**D**), as well as on the CD3^+^ T cells (**F**), CD4^+^ and CD8^+^ T cells (**G**), CD4^+^IFN−γ^+^ Th1, CD8^+^IFN−γ^+^ CTL, CD4^+^IL−4^+^ Th2, CD4^+^IL−17^+^ Th17, and CD4^+^FoxP3^+^ Treg (**H**) were shown. Data are represented as means ± SD from n = 3–6 per group, representative of one of three independent experiments. Statistical significances of differences are determined by one-way ANOVA (**B** and **F**) and two-way ANOVA (**D**, **G**, and **H**). * *p* < 0.05, ** *p* < 0.01, *** *p* < 0.001, **** *p* < 0.0001.

Next, we investigated the cellular sources of FcγRI to identify its action targets during the chlamydial defense. According to reports, mFcγRI expression appears to be restricted on the surface of myeloid cells including monocytes, macrophages, moDCs, and activated neutrophils [9,10,11]. To clarify the expression pattern of FcγRI on individual immune cell compartments following chlamydial infection, a multicolor flow cytometry strategy was employed (Supplementary Figure S1). As expected, raised expression of FcγRI on the CD11b^+^ myeloid cells was detected over the time-course of *C. muridarum* infection (Figure 2A,B). FcγRI levels on specific myeloid cell populations were then determined (Figure 2C,D). We observed that FcγRI expressed on the Ly6C^hi^ monocytes, Ly6C^lo^ monocytes, and macrophages at a basic level, while chlamydial infection induced significantly higher expression over the time-course of infection. As reported, we hardly detected expression of FcγRI on resting neutrophils and DCs; however, after infection, the expression level of FcγRI increased.

The inducible FcγRI expression on chlamydia-infected neutrophils and DCs encouraged us to detect FcγRI in more cell populations, such as T cells, which play vital roles in protection against chlamydial invasion. Surprisingly, we uncovered elevated FcγRI on CD3^+^ T cells and its CD4^+^ T cell subsets following infection (Figure 2E–G). Thus, we monitored FcγRI levels on effector T cell subsets including CD4^+^IFN−γ^+^ Th1, CD8^+^IFN−γ^+^ CTL, CD4^+^IL−4^+^ Th2, CD4^+^IL−17^+^ Th17, and CD4^+^FoxP3^+^ Treg (Figure 2H). The increased expression on the CD4^+^IFN−γ^+^ Th1 cells was indicated. These data revealed an unpredicted expression pattern of FcγRI on immune cell populations, implicating its regulation of innate and adaptive immunity against chlamydial infection.

### 3.3. Deficiency of FcγRI Differently Regulates the Recruitments of Innate and Adaptive Immune Cells Following C. Muridarum Infection

**Figure 3:**
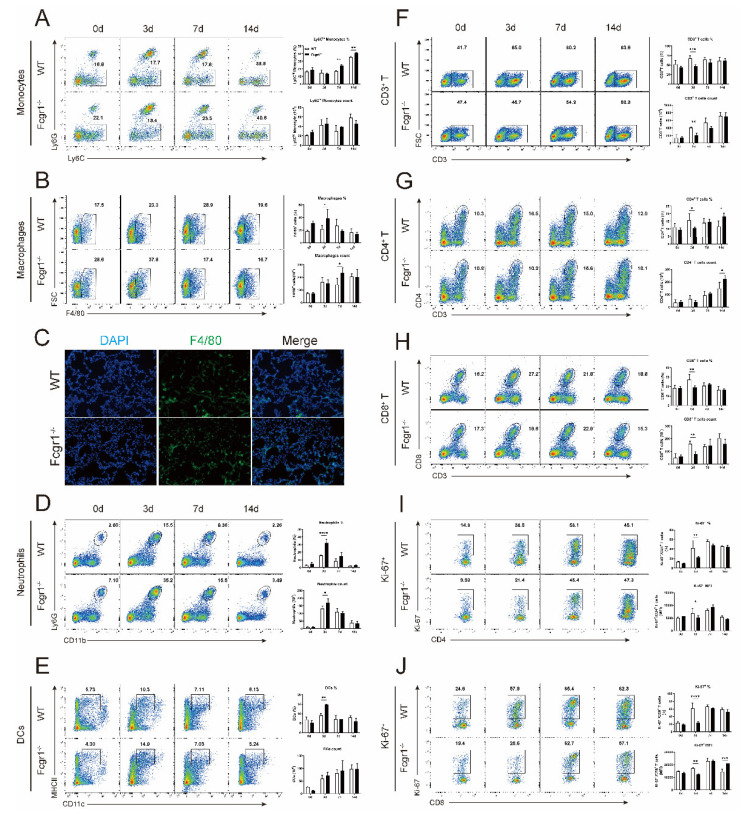
Altered numbers of innate and adaptive immune cells in Fcgr1^−/−^ mice after *C. muridarum* infection. The lung single cells of WT and Fcgr1^−/−^ mice on days 0, 3, 7, and 14 p.i. were prepared, and the infiltrations of innate and adaptive immune cells (gated as described in Supplementary Figure S1) were examined by flow cytometry. Representative flow cytometric plots (**left panel**), percentages (**top right panel**), and total numbers of (bottom right panel) of Ly6C^hi^ monocytes (**A**), macrophages (**B**), neutrophils (**D**), DCs (**E**), CD3^+^ T cells (**F**), CD4^+^ T cells (**G**), CD8^+^ T cells (**H**), Ki−67^+^CD4^+^ T cells (**I**) and Ki−67^+^CD8^+^ T cells (**J**) are shown. (**C**) The lung cryosections at day 3 p.i. were stained by anti-F4/80 for macrophages (green), and nuclei were stained with DAPI (blue), representative immunostaining captured by fluorescence microscopy in ×200 magnification. Data are represented as means ± SD from n = 3–5 per group, representative of one of three independent experiments. Statistical significances of differences are determined by two-way ANOVA. * *p* < 0.05, ** *p* < 0.01, *** *p* < 0.001, **** *p* < 0.0001.

Given the increased FcγRI expression in the innate and adaptive immune cells following infection, we further examined its impact on the recruitment of these cell populations by flow cytometry and immunofluorescence. As indicated in Figure 3A–H, *C. muridarum* invasion into WT mice significantly attracted innate and adaptive immune cells to the lung, including Ly6C^hi^ monocytes (Figure 3A), F4/80^+^ macrophages (Figure 3B), Ly6G^+^ neutrophils (Figure 3D), CD11c^+^MHCII^+^ DCs (Figure 3E), and CD3^+^ T cells (Figure 3F) (including CD4^+^ and CD8^+^ subsets in the Figure 3G and H, respectively). *C. muridarum* invasion into Fcgr1^−/−^ mice also increased immune cells in the lung compared with uninfected Fcgr1^−/−^ controls. However, compared with infected WT controls, *C. muridarum*-infected Fcgr1^−/−^ mice displayed increased innate immune cells (including monocytes, macrophages, neutrophils, and DCs) and reduced adaptive immune cells (CD3^+^ T cells and its CD4^+^ and CD8^+^ subsets), especially in the early infection (Figure 3A–H). In line with Figure 3B, immunofluorescence analysis also demonstrated more F4/80^+^ cell distribution in Fcgr1^−/−^ mice at day 3 p.i. (Figure 3C). Ki−67 staining showed lower proliferation of CD4^+^ and CD8^+^ T cells in Fcgr1^−/−^ mice at day 3 p.i. (Figure 3I–J). In the late infection, wherein Fcgr1^−/−^ mice exhibited milder disease, it is not neglectable that FcγRI deficiency recruited more CD4^+^ T cells (Figure 3G). The consistency between the alleviated disease and increased CD4^+^ T cells of the Fcgr1^−/−^ mice in the late infection hinted that CD4^+^ T cells might act as a main regulatory target of FcγRI.

### 3.4. Deficiency of FcγRI Promotes Th1 Response against C. muridarum Infection

**Figure 4:**
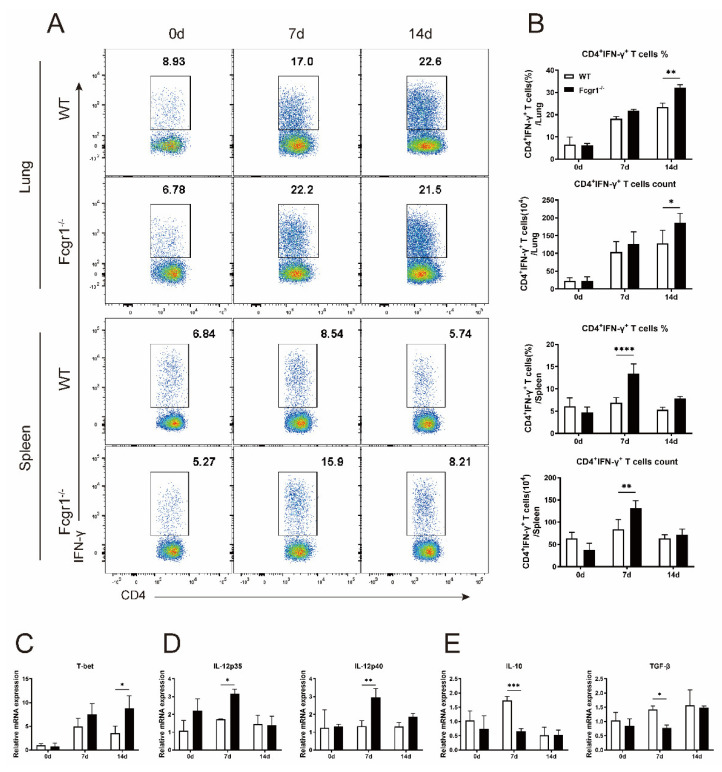
Increased Th1 response in Fcgr1^−/−^ mice after *C. muridarum* infection. (**A**,**B**) The lung and spleen single cells of WT and Fcgr1^−/−^ mice were prepared at days 0, 7, and 14 p.i. and the Th1 response was examined by flow cytometry. Representative flow cytometric plots of IFN-γ-producing CD4^+^ Th1 cells (**A**), summaries of their percentages and total numbers (**B**) are shown. (**C**–**E**) Total RNA extracted from the lung tissue was assayed for T−bet (**C**), IL−12p35/IL−12p40 (**D**), and IL−10/TGF−β (**E**) mRNA expression by qPCR. Data are represented as means ± SD from n = 3–5 per group, representative of one of three independent experiments. Statistical significances of differences are determined by two-way ANOVA. * *p* < 0.05, ** *p* < 0.01, *** *p* < 0.001, **** *p* < 0.0001.

As widely reported, *C. muridarum*-induced Th1 response promotes chlamydial clearance through the production of IFN-γ [17,18]. We uncovered the regulation of FcγRI on CD4^+^ T cells in the above results, combined with the elevated FcγRI expression on CD4^+^IFN-γ^+^ Th1 cells in Figure 2H, and we further compared the *C. muridarum*-driven Th1 response in Fcgr1^−/−^ and WT mice. The flow cytometry analysis showed that both the pulmonary and splenic CD4^+^T cells from Fcgr1^−/−^ mice produced significantly higher levels of IFN-γ (Figure 4A,B), reflecting increased Th1 responses in the local and systematic immune system. The T−box transcription factor TBX21 (T−bet), specific transcription factors of Th1 cells, showed increased mRNA expression in the lungs of Fcgr1^−/−^ mice (Figure 4C). The elevation in T−bet expression prompted us to investigate whether the enhanced differentiation into Th1 cells extends to the higher Th1 cytokines response. As predicted, compared with *C. muridarum*-infected WT mice, the mRNA expression of IL−12p35 and IL−12p40 (Th1-promoting cytokines) in Fcgr1^−/−^ mice showed a marked increase (Figure 4D). Furthermore, Th1-inhibitory cytokines IL−10 and TGF−β showed reduced expression levels in Fcgr1^−/−^ mice (Figure 4E). All these results revealed the potency of FcγRI in inhibiting the protective Th1 response, accounting for the improved chlamydial clearance in Fcgr1^−/−^ mice.

### 3.5. Deficiency of FcγRI Restricts the Macrophage Polarization toward M1 Phenotype during C. muridarum Infection

**Figure 5:**
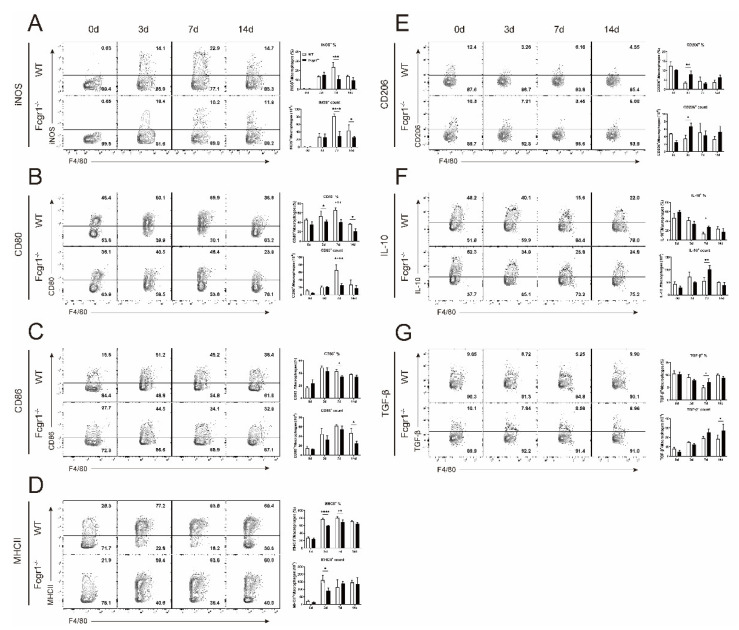
Restricted macrophage polarization towards M1 in Fcgr1^−/−^ mice after *C. muridarum* infection. The lung single cells of WT and Fcgr1^−/−^ mice on days 0, 3, 7, and 14 p.i. were prepared, and the macrophage polarization was examined by flow cytometry. Representative flow cytometric plots (left panel), percentages (top right panel), and total numbers (bottom right panel) of iNOS^+^ macrophages (**A**), CD80^+^ macrophages (**B**), CD86^+^ macrophages (**C**), MHCII^+^ macrophages (**D**), CD206^+^ macrophages (**E**), IL−10^+^ macrophages (**F**), and TGF−β^+^ macrophages (**G**) are shown. Data are represented as means ± SD from n = 3–5 per group, representative of one of three independent experiments. Statistical significances of differences are determined by two-way ANOVA. * *p* < 0.05, ** *p* < 0.01, *** *p* < 0.001, **** *p* < 0.0001.

We further explored the mechanism for FcγRI in exacerbating the inflammatory pathological response in chlamydial infection. Macrophages play an important role in the regulation of inflammation and related pathologies by altering polarization phenotypes [19,20,21]. Generally, polarized macrophages can be divided into classically activated macrophages (M1) and alternatively activated macrophages (M2). M1 macrophages are proinflammatory and up-regulate the expression of inducible Nitric oxide synthase (iNOS), costimulatory molecules CD80 and CD86, and MHC class II (MHCII), while M2 macrophages are anti-inflammatory, express CD206, and produce anti-inflammatory cytokines such as IL−10 and TGF−β [20]. As indicated in Figure 5A–G, following infection, pulmonary macrophages of WT mice expressed increased M1 markers and reduced M2 markers over the time-course of infection, indicating a pro-inflammatory effect of macrophages during chlamydial infection. Pulmonary macrophages in the lung of *C. muridarum*-infected Fcgr1^−/−^ mice also polarized to M1 phenotype compared with uninfected Fcgr1^−/−^ control. When compared to infected WT mice, the macrophages from the Fcgr1^−/−^ mice display reduced M1 and elevated M2 polarization. These findings indicated that FcγRI could promote macrophage polarization toward the proinflammatory M1 subtype, thus aggravating the pulmonary pathology during *C. muridarum* infection.

## 4. Discussion

In this research, we observed reduced bacterial loads and pulmonary pathology in Fcgr1^−/−^ mice following chlamydial respiratory infection, suggesting the regulation of FcγRI in bacterial clearance and inflammatory pathology. Further analysis revealed increased FcγRI levels in the immune system, especially in the macrophages and CD4^+^ Th1 cells. Compared with infected WT control, chlamydial infection elicited increased myeloid cells and reduced lymphocyte cells in early infection, as well as increased CD4^+^ T cells in late infection in Fcgr1^−/−^ mice. More importantly, enhanced Th1 response and constrained M1 macrophage polarization were observed in Fcgr1^−/−^ mice. Collectively, these findings suggested that FcγRI might play a pathological role by restricting protective Th1 response and aggravating macrophage proinflammatory polarization in chlamydial infection.

Based on the involvement of FcγRI in the immune response, research has described FcγRI’s contributions to the development and progression of several infections and inflammatory diseases. In 2002, Ioan-Facsinay A et al. revealed the regulation of FcγRI on the killing capacity by macrophages and MHC I-restricted presentation of IC-derived peptides by BMDC. In FcγRI^−/−^ mice, they further observed less joint swelling and reduced cartilage destruction in the experimental antigen-induced arthritis (AIA) model, and impaired bacterial clearance against *Bordetella pertussis* infection [13]. Nadine Barnes et al. demonstrated that FcγRI^−/−^ mice displayed reductions in both the kinetics and the magnitude of the Arthus reaction [12]. During PPRSV infection, Peidian Shi et al. identified six different FcγRI isoforms and uncovered dual regulation of these FcγRI splice variants, with the transcript 1 could promote endocytosis of the PRRSV-antibody complex to enhance PRRSV replication, and soluble transcript 3 showed an inflammation enhancement effect [14]. During a secondary chlamydial genital infection, combined with FcγRIII and FcγRIIB1, FcγRI has been shown to promote the killing capacity and antibody-dependent cell-mediated cytotoxicity (ADCC) of macrophages [22]. This study, which initially placed emphasis on the role of FcγRI in chlamydial respiratory infection, will contribute new theories to the field of FcγRI research in infectious and inflammatory illnesses.

As target cells of FcR, NK cells are one of the key components of innate immunity to invading pathogens [23,24,25,26]. In determining the contribution of FcγRI to host defense against chlamydial infection, we also observed potential modulation of FcγRI on this cell population. As indicated in Supplementary Figure S2, *C. muridarum* infection elicited increased FcγRI expression on the surface of the CD3^-^NK1.1^+^ NK cells both in the lung and spleen. Furthermore, we detected increased NK cells with higher CD69 expression (representing higher NK cell activity) in Fcgr1^−/−^ mice compared with WT controls. These data indicated that FcγRI might limit the infiltration and activation of NK cells in chlamydial infection. In the studies of chlamydial respiratory infection, NK cells were primarily defined to play important regulatory roles on other immune cells, such as DCs, Th1/Th17 cells, and macrophages [27,28,29], instead of directly killing the pathogen. Combined with the identified FcγRI’s impact on macrophages and Th1 response in this study, we believed that the FcγRI-regulated NK-macrophage interaction or NK-Th1 interaction will provide a promising direction for further mechanistic exploration.

Macrophage polarization was reported to regulate the magnitude of Th cell response. Generally, the M1 macrophages are involved in eliciting Th1 and Th17 responses, while M2 macrophages act as key effectors in Th2 responses [30,31]. The M1 and M2 macrophages orchestrate the Th cell response differently by secreting distinct chemokine and cytokine profiles: M1 macrophages recruit Th1 cells by producing Th1-recruiting chemokines CXCL9 and CXCL10, and Th1- and Th17-polarizing cytokines IL-12, IL-23, and IL-27, while M2 macrophages recruit Th2 cells and Tregs by chemokines CCL17, CCL22, and CCL24 [20,32,33]. In this study, chlamydial infection induced protective Th1 response and macrophage polarization toward M1; however, deficiency of FcγRI elicited enhanced Th1 response with reduced M1 macrophage polarization (Figure 5). We attributed this inconsistency to the diversity of Th1 differentiation factors. Indeed, the strength of T-cell receptor elicits (TCR) signaling, polarizing cytokines-mediated signals, cytokine-mediated positive feedback mechanism, lineage-specific master transcription factors, and the cross-regulation between different lineages both have critical impacts on Th cell-fate determination [34,35,36]. Our research here determined the transcription and cytokine level of Th1 response, and focused on the pathological impact of polarized macrophages (Figure 4 and Figure 5), preliminarily highlighting FcγRI’s modulation on chlamydial defense and inflammatory response. Further studies including multiple regulation pathways of FcγRI on Th1 response, as well as macrophage-T cell coculture experiments, are helping to improve our investigations.

The gene-knockout mice and blocking antibodies are two common strategies used to achieve the loss-of-function of target genes in many in vivo disease and therapy models. In this study, using FcγRI-deficient mice, we determined the contribution of FcγRI on the immune response to chlamydial infection. Specifically, we tried to find the neutralization antibody for specifically blocking the function of mFcγRI, and met serious limitations. In a review in 2015, Bruhns P et al. stated that no blocking antibody against mFcγRI was available till then [9]. By reviewing the literature of recent years, we still have not found an available blocking antibody specific for FcγRI. Despite the Ultra-LEAF™ Purified anti-mouse CD64 (FcγRI) Antibody (Biolegend, 941904), no evidence has been provided to date for its application to in vivo experiments or published investigations.

## 5. Conclusions

In summary, our study advances the emerging knowledge of the roles of FcγRI in inflammatory and respiratory diseases. The discovery of the regulation and immune mechanism of FcγRI on the innate and adaptive immunity may facilitate the development of novel therapeutics in chlamydial infectious diseases.

## Data Availability

The raw data used to support the findings of this study are available from the corresponding author upon request.

## Supplementary Materials

The following supporting information can be downloaded at: https://www.mdpi.com/article/10.3390/microorganisms11010039/s1. Table S1: The primer sequences used for qPCR analysis; Figure S1: The flow cytometry gate strategy for the analysis of innate and adaptive immune cells; Figure S2: Infiltrations and activation of NK cells in the lung and spleen of Fcgr1^−/−^ mice following *Chlamydia muridarum* (*C. muridarum*) respiratory infection.
