# Supplementary material for: A Pathogenic Role for FcγRI in the Immune Response against Chlamydial Respiratory Infection
Source: Microorganisms. 2022 Dec 22;11(1):39. doi: 10.3390/microorganisms11010039 (PMC9862693; doi:10.3390/microorganisms11010039)
Supplement: Supplementary file 1 [file microorganisms-11-00039-s001.zip › microorganisms-2088925-supplementary.pdf]

A pathogenic role for FcγRI in the immune response against chlamydial respiratory infection

Supplementary Table S1. The primer sequences used for qPCR analysis.

Gene name	Forward primer (5’→3’)	Reverse primer (5’→3’)
β-actin	GGCTGTATTCCCCTCCATCG	CCAGTTGGTAACAATGCCATGT
Fcgr1	TCCACACAATGGTTTATCAACGG	CACTGTCCTGAAAACTGGCCT
16S rRNA	CGCCTGAGGAGTACACTCGC	CCAACACCTCACGGCACGAG
IL-6	TGAACAACGATGATGCACTTGCAG	TAGCCACTCCTTCTGTGACTCCAG
IL-1β	GAAATGCCACCTTTTGACAGTG	TGGATGCTCTCATCAGGACAG
TNFα	CTGAACTTCGGGGTGATCGG	GGCTTGTCACTCGAATTTTGAGA
T-bet	AACCGCTTATATGTCCACCCA	CTTGTTGTTGGTGAGCTTTAGC
IL-12p35	CAATCACGCTACCTCCTCTTTT	CAGCAGTGCAGGAATAATGTTTC
IL-12p40	TGGTTTGCCATCGTTTTGCTG	ACAGGTGAGGTTCACTGTTTCT
IL-10	CTTACTGACTGGCATGAGGATCA	GCAGCTCTAGGAGCATGTGG
TGF-β	AAAACAGGGGCAGTTACTACAAC	TGGCAGATATAGACCATCAGCA

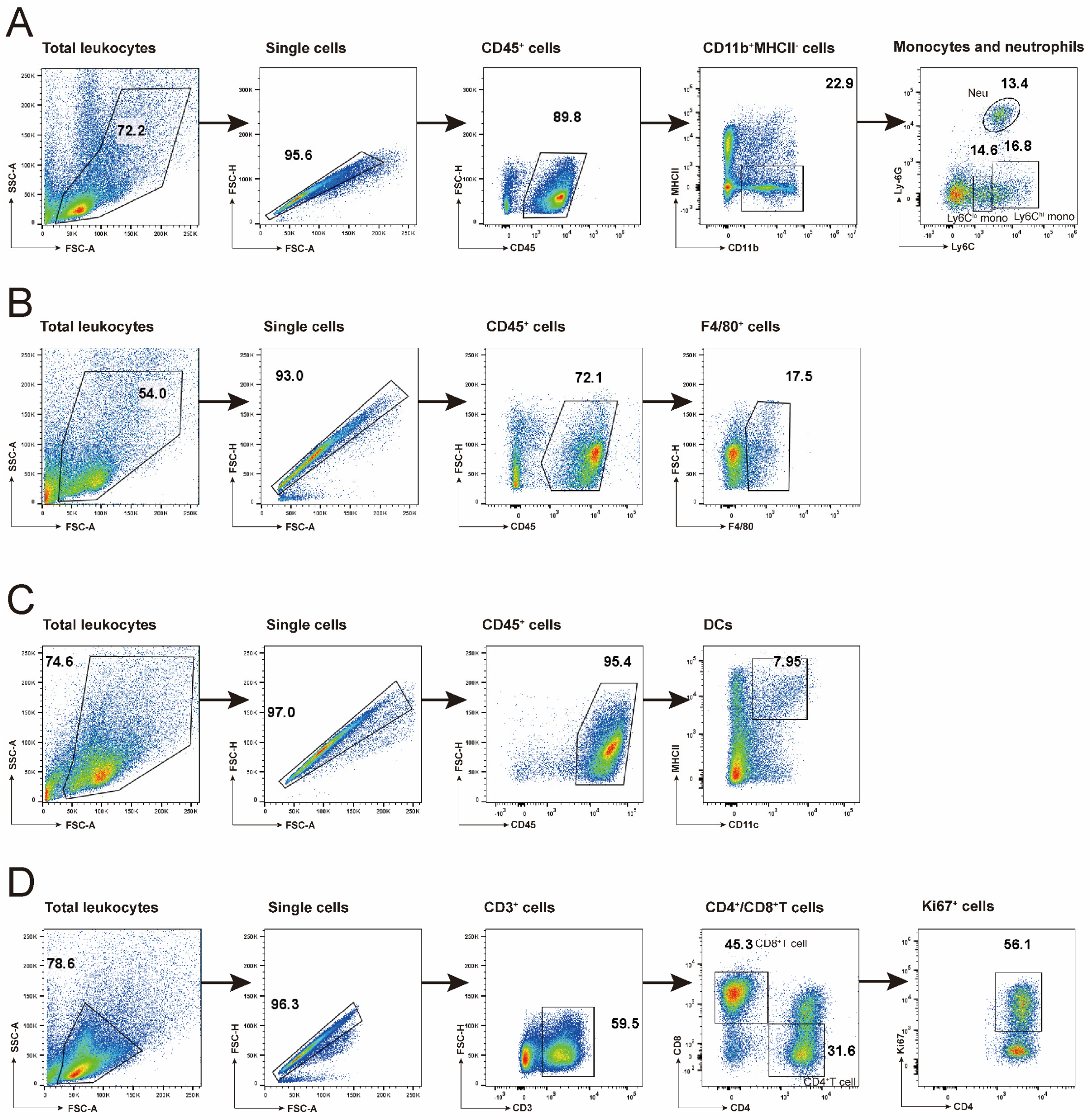

Supplementary Figure S1. The flow cytometry gate strategy for the analysis of innate and adaptive immune cells. Related to Figures 2 and 3. (A) Ly6C^{hi} monocytes were presented as CD45^{+}CD11b^{+}MHCII^{-}Ly6G^{-}Ly6C^{hi} cells, Ly6C^{lo} monocytes were presented as CD45^{+}CD11b^{+}MHCII^{-}Ly6G^{-}Ly6C^{lo} cells, and neutrophils were presented as CD45^{+}CD11b^{+}MHCII^{-}Ly6G^{+} cells. (B) macrophages were presented as CD45^{+}F4/80^{+} cells. (C) DCs were presented as CD45^{+}CD11c^{+}MHCII^{+} cells. (D) CD4^{+} T cells were presented as CD3^{+}CD4^{+} cells, and CD8^{+} T cells were presented as CD3^{+}CD8^{+} cells.

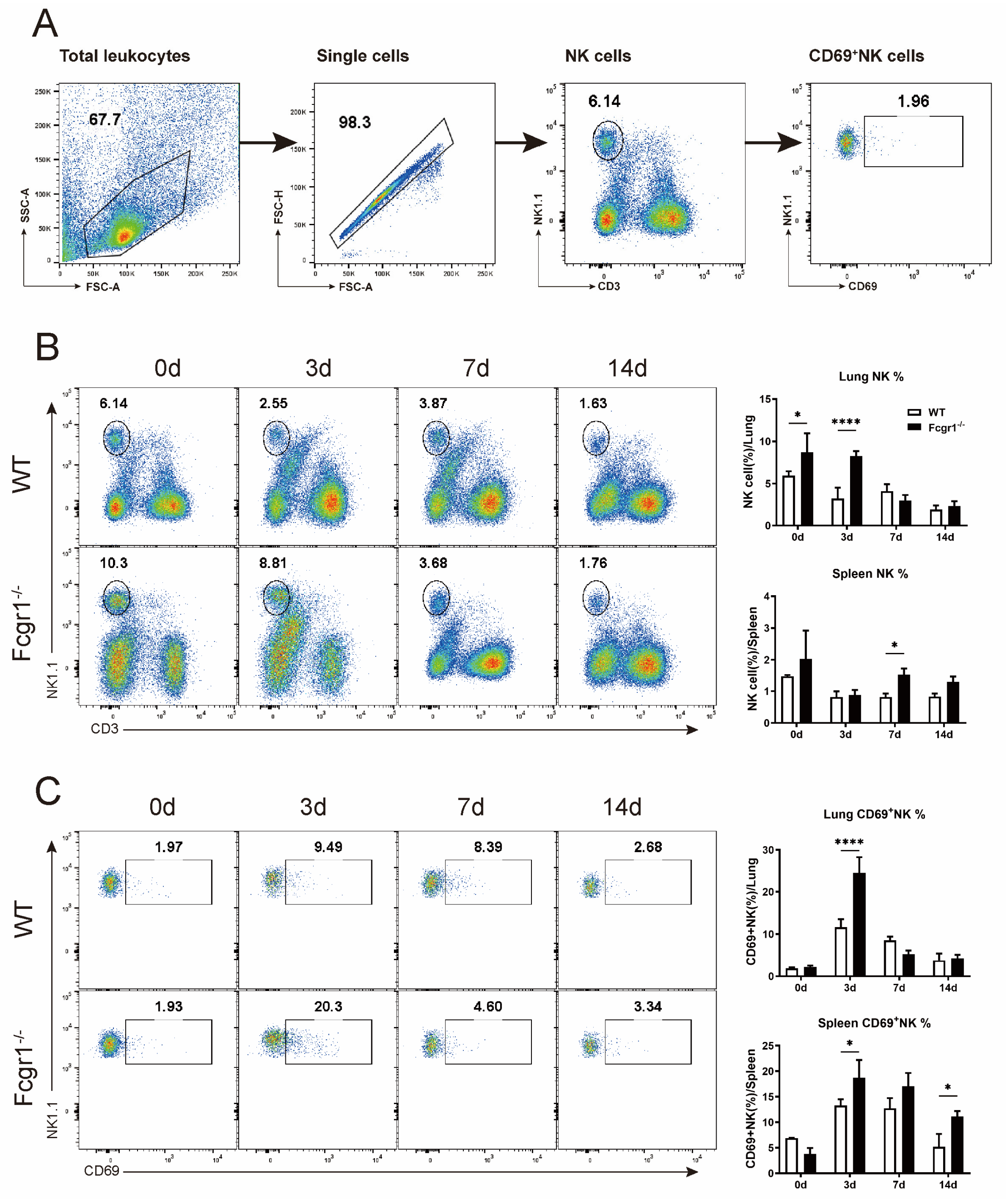

Supplementary Figure S2. Infiltrations and activation of NK cells in the lung and spleen of Fcgr1$^{-/-}$ mice following *Chlamydia muridarum* (*C. muridarum*) respiratory infection. An animal model of *C. muridarum* respiratory infection was induced by intranasally inoculating with 1×10^{3} inclusion forming units (IFUs) *C. muridarum*. The lung and spleen single cells of wild-type (WT) and FcγRI deficient (Fcgr1$^{-/-}$) mice on days 0, 3, 7, and 14 post-infection (p.i.) were prepared. (A) Representative flow cytometry gating to identify NK cells (CD3^{-}NK1.1^{+} cells) and the activity of NK cells (CD3^{-}NK1.1^{+}CD69^{+} cells). (B) Representative flow cytometric plots (left panel) and

the percentages of NK in the lung (top right panel) and spleen (bottom right panel) are shown. (C) Representative flow cytometric plots (left panel) and the percentages of $CD69^+$NK cells in the lung (top right panel) and spleen (bottom right panel) are shown. Data are represented as means ± SD from n = 3 - 5 per group, representative of one of three independent experiments. Statistical significances of differences are determined by two-way ANOVA followed by Šídák's multiple comparisons test. * $P < 0.05$, **** $P < 0.0001$.
